# The Use of Tricaine Methanesulfonate (MS-222) in Asian Seabass (*Lates calcarifer*) at Different Temperatures: Study of Optimal Doses, Minimum Effective Concentration, Blood Biochemistry, Immersion Pharmacokinetics, and Tissue Distributions

**DOI:** 10.3390/vetsci10090539

**Published:** 2023-08-24

**Authors:** Julia Chu-Ning Hsu, Tirawat Rairat, Yi-Ping Lu, Chi-Chung Chou

**Affiliations:** 1 Department of Veterinary Medicine, College of Veterinary Medicine, National Chung Hsing University, No. 145, Xingda Rd., South Dist., Taichung 40227, Taiwan; juliacnhsu@dragon.nchu.edu.tw; 2 Department of Fishery Biology, Faculty of Fisheries, Kasetsart University, 50 Paholyotin Rd., Ladyao, Chatujak, Bangkok 10900, Thailand; ffistwr@ku.ac.th; 3 Biology Division, Veterinary Research Institute, Ministry of Agriculture, Executive Yuan, No. 376, Zhongzheng Rd., Danshui Dist., New Taipei City 25158, Taiwan; yplu@mail.nvri.gov.tw

**Keywords:** anesthesia, anesthetic agent, fish, aquaculture, animal welfare

## Abstract

**Simple Summary:**

The optimal use of tricaine methanesulfonate (MS-222) in Asian seabass under different rearing temperatures was not established, and the mechanisms behind the optimal use were not known. This study aimed to determine the optimal doses and minimum effective concentrations (MECs) of MS-222 in Asian seabass reared at 22 and 28 °C. Serum biochemistry, pharmacokinetics, and tissue distributions of MS-222 following immersion at the determined optimal doses were evaluated to delineate possible mechanisms dictating the temperature difference. The results showed that water temperature exerted no or minimal impact on the optimal doses and the MECs. Fish exposed to the optimal doses had significantly elevated blood concentrations of lactate, glucose, calcium, magnesium, and sodium, while the blood pH was significantly decreased. The fish eliminated MS-222 two times faster at 28 °C than at 22 °C. Tissue-specific distribution patterns were observed. Irrespective of water temperature, MS-222 peaked earliest in the brain and gill (5 min), followed by liver and kidney (10–20 min). Most tissues exhibit a gradual decline of drug concentration except for the gill, which was maintained at a steady level. Muscle is the least perfused tissue with the lowest drug concentration. This study provided evidence contributing to a better understanding of the actions of MS-222 in Asian seabass at different temperatures.

**Abstract:**

This study was conducted to determine the optimal doses and minimum effective concentrations (MECs) of tricaine methanesulfonate (MS-222) in marketable-size Asian seabass reared at two temperatures (22 and 28 °C). Serum biochemical parameters, pharmacokinetics, and tissue distributions of MS-222 following immersion at the determined optimal doses were also evaluated in order to delineate possible mechanisms dictating the temperature difference. The definition of optimal dose is set as the dose when fish attain stage III anesthesia within 5 min, sustain this stage for 3 min, and re-attain equilibrium within 5 min. The MEC is the fish serum MS-222 concentration when stage III anesthesia is reached. The results showed that water temperature exerted no or minimal impact on the designated parameters. The optimal doses at 22 and 28 °C were 140 and 150 µg/mL, while the MECs were 70.48 and 78.27 µg/mL, respectively. Fish exposed to the optimal doses of MS-222 had significantly elevated blood concentrations of lactate, glucose, calcium, magnesium, and sodium, while the blood pH was significantly decreased. The fish eliminated MS-222 faster at 28 °C than at 22 °C, with serum half-lives of 18.43 and 37.01 h, respectively. Tissue-specific distribution patterns were evident. Irrespective of water temperature, MS-222 peaked at 5 min for the brain and gill but peaked slightly later at 10–20 min for the liver and kidney. Most tissues exhibit a gradual decline of drug concentration except for the gill, which was maintained at a steady level. Muscle is the least perfused tissue with the lowest drug concentration throughout the 90 min period. This study provided physiological and pharmacokinetic evidence contributing to a better understanding of the actions of MS-222 in Asian seabass at different temperatures.

## 1. Introduction

The usage of anesthetics is eminent in aquaculture for improving fish welfare and facilitating various routine procedures via reducing stress during transportation, handling, sorting, blood sampling, and surgery [1,2]. Among various anesthetics used in fish, tricaine methanesulfonate (MS-222) is one of the most common agents suitable for use in both seawater and freshwater fish [3] and has a rapid effect in reducing nerve and muscular activity [4]. According to the United States Food and Drug Administration (US FDA), MS-222 is the only approved anesthetic for food fish and has a withdrawal time of 21 days [5]. In Taiwan, in addition to eugenol, MS-222 is also approved for grouper with a withdrawal time of 5 days [6].

Fish are a poikilothermic animal in which rearing temperature has confounding effects on its physiological conditions [7]. In general, higher water temperature has shortened induction and recovery phases of the anesthetics [8,9]. However, it has been demonstrated that pikeperch (*Sander lucioperca*) at 20 and 23 °C require a similar dosage (100 µg/mL) of MS-222 to reach stage III anesthesia [9]. Likewise, sailfin molly (*Peocilia latipinna*) at 20 and 30 °C require a similar level of MS-222 concentration (150 µg/mL) [10]. These observations indicate that the appropriate dosages of MS-222 may vary among different fish species.

After immersion with MS-222, the drug can pass the blood–brain barrier and be distributed to the central nervous system [9,11]. MS-222 is believed to produce the state of anesthesia by blocking the sodium channels of the neurons [12] and preventing the initiation of the action potential to inhibit the subsequent sensory communication between the brain and the motor system [13]. The cardiovascular system is also affected; tachycardia is observed within 30 sec after MS-222 administration, which is a result of the immediate effect on central nervous system [14]; bradycardia then follows, presumably resulting from the direct effect of MS-222 on the myocardium [14,15].

The US FDA recommended dose of MS-222 ranges from 10 to 1000 µg/mL [5]. In Taiwan, the recommended dose of MS-222 is 30 µg/mL for anesthesia in grouper [6]. Although the general recommended dose range of MS-222 is usually broad; in practice, an optimal dose that is effective and safe for a given species under a specific environmental condition should be preferred. The reported optimal doses of MS-222 under a specified rearing condition are 70–90 µg/mL for Asian seabass (*Lates calcarifer*) [16], 75 µg/mL for fathead minnow (*Pimephales promelas*) [17] and Senegalese sole (*Solea senegalensis*) [18], 90–110 µg/mL for yellow catfish (*Tachysurus fulvidraco*) [19], 100 µg/mL for silver seabream (*Pagrus auratus*) [20], 100–200 µg/mL for lumpfish (*Cyclopterus lumpus*) [21], and 300 µg/mL for Nile tilapia (*Oreochromis niloticus*) [22] and goldfish (*Carassius auratus*) [23].

The minimum effective concentration (MEC) is conventionally defined as the anesthetic concentration in the blood when the fish reached stage III anesthesia, and it is believed to be an important piece of supporting evidence for proper anesthetic use [22]. The MEC values of MS-222 also vary greatly in different fish species. For example, the MECs of 100 µg/mL or higher are reported in gars (*Lepisosteus platostonus* and *L. osseus*), bowfin (*Amia calva*), channel catfish (*Ictalurus punctatus*), and bluegill (*Lepomis macrochirus*) [11]. In comparison, northern pike (*Esox lucius*), rainbow trout (*Oncorhynchus mykiss*), silver seabream, and Nile tilapia have lower MECs, usually around 70–80 µg/mL [11,20,22], whereas that of Atlantic salmon (*Salmo salar*) is only 16 µg/mL [24].

Asian seabass is an important aquaculture species in the Indo-West Pacific region with high economic value. Even though the optimal dose of MS-222 for anesthesia in Asian seabass (200 g) has been previously reported as 70–90 µg/mL at 25 °C [16], the effects of water temperature on the optimal dose and MEC were still unknown. Furthermore, the pharmacokinetics, tissue distributions, and blood biochemistry information, which may contribute to a greater understanding of the physiological–chemical interplay of the drug in the body remain to be elucidated. Therefore, the aims of this study were to determine the optimal doses and MECs of MS-222 in Asian seabass with consideration of the temperature effect. The changes in blood biochemical parameters, in combination with the pharmacokinetics (PK) and tissue distributions of MS-222 were also investigated to delineate any possible mechanisms. This study shall provide greater insight into the rational use of MS-222 in Asian seabass.

## 2. Materials and Methods

### 2.1. Chemicals and Preparation of MS-222 Solutions

MS-222 (analytical grade), sodium bicarbonate (NaHCO_3_), and sodium dihydrogen phosphate (NaH_2_PO_4_) were purchased from Sigma-Aldrich (St. Louis, MO, USA). Disodium hydrogen phosphate (Na_2_HPO_4_) was obtained from PanReac AppliChem (Barcelona, Spain). Acetonitrile (MeCN; HPLC grade) was purchased from Avantor Performance Materials (Center Valley, PA, USA). The MS-222 solutions were freshly prepared before use by dissolving the MS-222 standard powder in double-distilled water to obtain the desired concentrations. The MS-222 was buffered with sodium bicarbonate at a ratio of 1:2 to ensure that the pH value was around 7 [3].

### 2.2. Experimental Fish

Asian seabass (350–550 g) from a commercial fish farm in Pingtung County, Taiwan, were brought to the Department of Veterinary Medicine, National Chung Hsing University, and kept in outdoor 1500 L tanks containing 5 ppt salt water at around 25 °C. In order to acclimate progressively to 22 or 28 °C, each individual fish was transferred to a 60 L tank that contained 5 ppt salt water for one week before conducting the experiment. Water temperatures were adjusted to the predetermined levels and maintained by aquarium heaters (Ming Zen Aquarium Company., Ltd., Taichung, Taiwan). The temperature (22 and 28 °C), dissolved oxygen (≥5.0 µg/mL), and pH (7.0–8.0) were checked daily with a portable water quality meter (WA-2017SD, Lutron Electronics Co., Inc., Coopersburg, PA, USA). All experimental protocols were approved by the Institutional Animal Care and Use Committee of National Chung Hsing University (No. 110-093). The animals were managed according to the Guidelines for the Care and Use of Laboratory Animals established by the Council of Agriculture, Executive Yuan in Taiwan.

### 2.3. Blood Collection

Before the MS-222 administration and as soon as the Asian seabass reached stage III anesthesia, the fish were taken out of the tank for blood collection. Blood samples (0.8 mL) were then collected from the caudal vessel without anticoagulant (22G needle attached to a 1 mL syringe). Half of the collected blood (0.4 mL) was placed into a lithium-heparin tube (BD Microtainer; Becton, Dickinson and Company, Franklin Lakes, NJ, USA) for the biochemistry analysis. The rest of blood samples were centrifuged at 1743× *g* for 10 min at room temperature. The supernatants (serum) were stored at −80 °C for later analysis of MS-222 concentrations to determine the MEC values.

### 2.4. Determination of the Optimal Doses after Single MS-222 Immersion

The optimal dose of MS-222 in this study is defined as the minimal dose that could induce stage III anesthesia in less than 5 min, sustain stage III for 3 min, and regain equilibrium within 5 min in the recovery tank as in our previous study [22]. For each temperature level, 21 fish were divided into 3 groups (*n* = 7), corresponding to the 3 doses of MS-222. The tested MS-222 levels at 22 °C were 100, 140, and 175 µg/mL, and those at 28 °C were 100, 150, and 200 µg/mL. The selection of these experimental doses was based on a preliminary study (see discussion). A dose that met all criteria mentioned above was considered an optimal dose and was chosen for the subsequent experiments (see below).

### 2.5. Determination of the Minimum Effective Concentrations (MECs) by HPLC

The MEC is the serum MS-222 concentration right after the fish reached stage III anesthesia. The serum samples were prepared following a previous study [22]. Briefly, 50 µL of serum was mixed with 150 µL of MeCN (containing 0.1% formic acid) solution in a microcentrifuge tube and centrifuged for 10 min at 1743× *g*. The supernatant was moved to a new tube and mixed with the MeCN solution to obtain a final volume of 200 μL for HPLC analysis.

The HPLC analysis was performed as previously described [22]. In brief, 60 μL of the above final supernatant was mixed with 140 μL of phosphate buffer (10 mM NaH_2_PO_4_-Na_2_HPO_4_, pH 5). After that, each sample was filtered through a 0.22 μm nylon filter before being injected into the HPLC system. The HPLC system consists of a pump (Waters 1525, Waters, Milford, MA, USA), a UV–visible detector (Waters 2489, Waters, Milford, MA, USA), and a C-18 column with 5 μm particle size and sized 150 × 4.6 mm (Apollo, Hichrom, UK). The mobile phase was a combination of MeCN and phosphate buffer (10 mM NaH_2_PO_4_-Na_2_HPO_4_, pH 5) (30:70). The injection volume was 50 μL, the flow rate was 1 mL/min, and the detection wavelength was 220 nm.

### 2.6. Establishment of the Calibration Curves

A stock solution of MS-222 (1000 μg/mL) was prepared in 70% MeCN. From this stock solution, subsequent dilutions were carried out to obtain different concentrations of working solutions. The working solutions of MS-222 were added into drug-free fish serum or homogenized tissues to obtain the final concentrations of 0.5, 1, 2.5, 5, 10, 50, and 100 μg/mL in the serum; 1, 5, 10, and 50 μg/g in the brain and liver; and 1, 2.5, 5, and 10 μg/g in the kidney, gill, and muscle. Then, the samples were extracted and analyzed by the above-mentioned HPLC method. At lower calibration concentrations, the weighting factor of 1/x^2^ was utilized to improve the accuracy of the calibration curve [25]. The LOD and LOQ were calculated by 3.3 × σ/S and 10 × σ/S, respectively (σ = standard deviation of the y-intercept of the regression line; S = slope of the calibration curve). However, if the calculated LOQ is below the lowest concentration point of the calibration curve, the lowest point of concentration will be used as the LOQ.

### 2.7. Assessment of Blood Biochemical Parameters

Heparinized blood samples of the fish exposed to the optimal doses MS-222 were subjected to biochemical analysis and venous blood gas test. Biochemical analyses including concentrations of lactate, glucose, blood urea nitrogen (BUN), calcium, magnesium, sodium, potassium, and chloride were performed with an AU480 Chemistry Analyzer (Beckman Coulter, Inc., Brea, CA, USA). Venous blood gas testing was performed with the VetStat Electrolyte Blood Gas Analyzer (IDEXX Laboratories, Inc., Westbrook, ME, USA). The analyzed values included pH, total carbon dioxide (tCO_2_), bicarbonate ion (HCO_3_^−^), and partial pressure of carbon dioxide (pCO_2_). All blood samples were analyzed within 20 min of sample collection at room temperature.

### 2.8. Pharmacokinetic Study

The pharmacokinetic (PK) study was performed by immersion administration of MS-222 at the determined optimal dose (140 µg/mL at 22 °C and 150 µg/mL at 28 °C). For each temperature level, 48 fish were immersed with the optimal dose for 3 min. Then, the fish were put into recovery tanks. Eight fish were sampled from the caudal vessels at each of the predetermined time points: 0, 5, 10, 20, 40, and 60 min. The blood samples were processed and subjected to the HPLC analysis as described above. Pharmacokinetic analysis of serum MS-222 was performed by a non-compartmental analysis using PKSolver 2.0 software (China Pharmaceutical University, Nanjing, China).

### 2.9. Tissue Distribution Study

For each temperature, 27 fish were immersed with the determined optimal dose of MS-222 (140 µg/mL at 22 °C and 150 µg/mL at 28 °C) until the fish reached stage III anesthesia. Then, the fish were put into recovery tanks. Five fish were randomly selected and sacrificed at the predetermined time points except for the last time point, in which only two fish were used (5, 10, 20, 40, 60, and 90 min). The fish were euthanized as suggested by the AVMA guidelines: by rapid severance of the head from the spinal cord and followed by pithing of the brain [26]. The visceral organs (brain, liver, gill, muscle, and kidney) were quickly removed and stored at −20 °C until analysis.

For tissue sample preparation, 150 mg of the homogenized brain in 1.5 mL microcentrifuge tube was mixed with MeCN containing 0.1% formic acid (450 µL) and centrifuged at 1743× *g* for 10 min. The supernatant was moved to a new microcentrifuge tube and MeCN with 0.1% formic acid was added to attain a final volume of 600 μL. For the kidney, gill, muscle, and liver, 1.0 g of homogenized tissue was mixed with MeCN containing 0.1% formic acid (3 mL) in a 5 mL tube and centrifuged at 2274× *g* for 10 min. The supernatant was moved to a new tube with addition of the MeCN solution to make a final volume of 4 mL. Lastly, the extracted tissue samples were analyzed by the HPLC method described above.

### 2.10. Statistical Analysis

All data are expressed as mean ± SD. The Prism 8 software (Graphpad Software Inc., San Diego, CA, USA) was used for all statistical analysis. For each temperature level, the statistical analysis of the optimal doses and MECs among different doses were evaluated through one-way ANOVA, followed by Tukey’s test for multiple comparisons. Differences in the biochemical parameters before and after MS-222 exposure and tissue concentrations between two temperatures were compared using independent *t*-test. Significant differences were considered at *p* < 0.05.

## 3. Results

### 3.1. Determination of Optimal Doses after Single MS-222 Immersion

The optimal dose was selected according to the behavioral criteria mentioned above. At 100 µg/mL MS-222, no fish could be induced to stage III anesthesia in less than 5 min. At the highest dose (200 µg/mL for 28 °C and 175 µg/mL for 22 °C), not all fish could recover within 5 min. Therefore, 140 µg/mL (at 22 °C) and 150 µg/mL (at 28 °C) were considered the optimal doses for their respective temperatures (Table 1). In both temperatures, the induction was inversely correlated with increasing MS-222 concentrations, whereas the recovery time appeared to be positively correlated.

### 3.2. Validation of the HPLC Method for Quantification of MS-222

The chromatogram of MS-222 showed no interference from endogenous components at the retention time (12 min). The matrix calibration curves were linear over the range of 0.50 to 50 μg/mL for the serum, 1.0 to 50 μg/g for the brain and liver, and 1.0 to 10 μg/g for the kidney, gill, and muscle with a weighted r^2^ of 0.9762, 0.9844, 0.9681, 0.9036, 0.8490, and 0.9828, respectively. For serum, brain, liver, kidney, gill, and muscle, the LODs were 117 ng/mL, 217, 312, 524, 676, and 212 ng/g, and the calculated extended LOQs were 355 ng/mL, 657, 946, 1547, 2049, and 644 ng/g, respectively. The practical LOQs used for data curation were 0.5 µg/mL, 1, 1, 1.5, 2.0, and 1 µg/mL, respectively. The extraction recoveries were above 80% for serum, brain, and muscle, while they were around 50% for liver and gill. The precisions shown as % CV were all less than 10%, except for some lower concentrations in the kidney and gill, which may reach 20%.

### 3.3. Determination of the Minimum Effective Concentration (MEC)

The MECs of different doses of MS-222 at each temperature level are shown in Table 1. The difference in the MEC values among 3 doses was not statistically significant. Thus, they were averaged to show a grand mean (70.48 µg/mL). Likewise, at 28 °C the MECs of the 100, 150, and 200 µg/mL doses (i.e., 76.84, 76.03, and 81.96 µg/mL) were similar; the grand mean at 28 °C was 78.27 µg/mL.

### 3.4. Assessment of Blood Biochemical Parameters

After immersion with MS-222, fish generally experienced an increase in blood lactate, glucose, calcium, magnesium, and sodium and a decrease in blood pH regardless of temperature (Table 2). Potassium and pCO_2_ were significantly decreased and increased, respectively, only at 22 °C, whereas chloride and anion gap were significantly increased only at 28 °C. The MS-222 immersion did not affect the levels of BUN, HCO_3_^−^, and tCO_2_.

### 3.5. Pharmacokinetic Study

**Figure 1:**
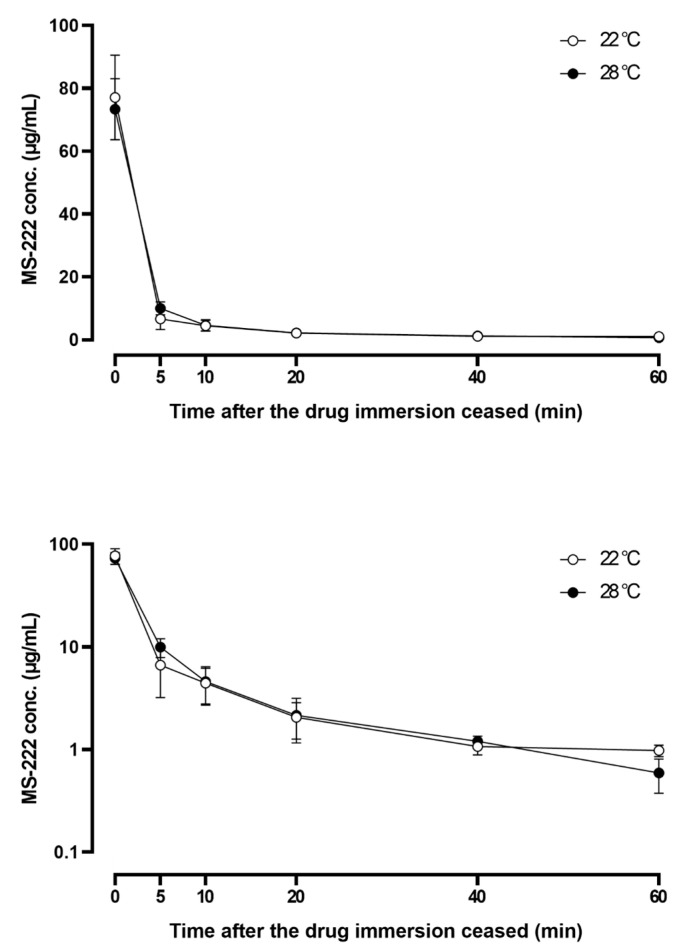
Linear (**upper**) and semi-logarithmic (**lower**) plots of the serum concentration–time profile of tricaine methanesulfonate (MS-222) after immersion at the optimal doses at 22 °C (140 µg/mL for 3 min) and 28 °C (150 µg/mL for 3 min). Data represented are mean ± SD (*n* = 8).

The depletion of MS-222 in serum after immersion with the optimal doses in the two temperatures is presented in Figure 1. The MS-222 concentration followed a biphasic decline, decreased by more than 80% in the first 5 min after the drug immersion ceased, and reduced by more than 95% in 20 min, suggesting quick distribution into the tissues (Figure 1, lower panel) and elimination. At the last blood sampling time (60 min), only about 1% of MS-222 remained in the serum. The depletion pattern at the two temperatures was very similar. The PK parameters are shown in Table 3. The t_1/2λ_ and MRT were twofold shorter at the warmer temperature. Nonetheless, the AUC at 22 °C (304.48 min·µg/mL) and 28 °C (306.68 min·µg/mL) was similar.

### 3.6. Tissue Distribution Study

**Figure 2:**
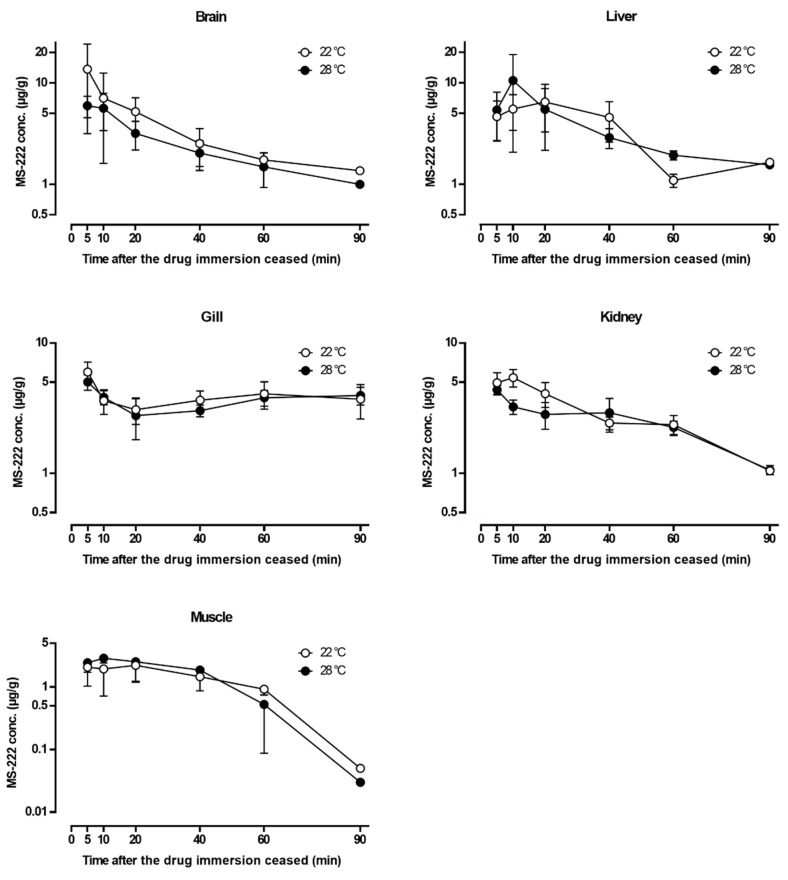
Semi-logarithmic plots of the tissue concentration–time profiles of tricaine methanesulfonate (MS-222) after immersion at the optimal doses at 22 °C (140 µg/mL) and 28 °C (150 µg/mL). Data are represented as mean ± SD (*n* = 5, except the last time point, in which *n* = 2). For muscle concentrations below 1 µg/mL, the extended estimation from the calibration curve was used to show the trend of decline.

**Figure 3:**
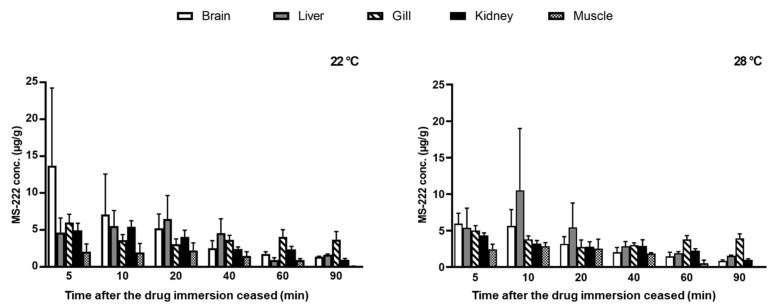
Tissue concentrations of tricaine methanesulfonate (MS-222) after immersion at the optimal doses at 22 °C (140 µg/mL) and 28 °C (150 µg/mL). Data are represented as mean ± SD (*n* = 5, except the last time point, in which *n* = 2).

The MS-222 was widely distributed into various visceral organs (Figure 2 and Figure 3). At 5 min after the fish was at stage III anesthesia, the MS-222 concentrations in most organs were around 4–6 µg/g except for the brain at 22 °C (13.7 µg/g); the muscle concentrations at both temperatures were about 2 µg/g and below. Generally, the MS-222 concentrations in the brain, liver, and kidney were progressively decreased with time, and at the last time point (90 min), the MS-222 in these three tissues was close to 1 µg/g. However, the drug concentrations in the gill were maintained at around 3–4 µg/g for most of the time despite an initial drop at 5–20 min, and the drug concentrations in the muscle were first stabilized at about 2 µg/g from 5–40 min and then rapidly declined below the LOQ level at 90 min. It is interesting to note that the MS-222 concentrations in the liver (at both temperatures), kidney (at 22 °C), and muscle (at 28 °C) actually increased after 5 min and reached a peak at either 10 or 20 min. The effect of water temperature on the distribution of MS-222 was not obvious since the difference in the MS-222 concentration in each tissue between the two temperatures was not statistically significant. Moreover, it should be mentioned that the experimental doses at 22 and 28 °C were different, so the temperature effect on the tissue concentrations of MS-222 could not be unequivocally demonstrated unless the same dose of MS-222 was used (see discussion).

## 4. Discussion

The efficacy of anesthetic agents in aquatic species is affected by biotic and abiotic factors, such as size, age, sex, salinity, pH, oxygen level, and water temperature [24,27,28,29,30]. Among these factors, temperature is arguably the most critical one [7,30]. The effect of rearing temperature can be observed in the induction time in the present study; following 100 µg/mL MS-222 administration, Asian seabass needed a twofold longer induction time at 28 °C than at 22 °C. This finding was unexpected as most studies reported the opposite direction, i.e., shortening the induction time as the temperature increased [8,21,29,31]. In general, higher temperature speeds up the drug absorption rate such that the drug concentration reaches MEC earlier, thereby reducing the induction time. However, this seemingly paradoxical result was supported by the fact that brain MS-222 concentration at 22 °C is much higher than at 28 °C. It might also be because the drug elimination rate is usually accelerated at warmer temperatures as well [22,32,33,34]. The interaction between the drug absorption and elimination rates at a particular temperature level will determine the overall fate of the drug in the fish body. Nonetheless, water temperature appeared to exert no discernible effect on the MECs (i.e., about 70–80 µg/mL) and optimal doses (i.e., 140–150 µg/mL) under the current experimental setting. In the optimal dose determination, our dose selections (i.e., 100, 140, and 175 µg/mL at 22 °C; 100, 150, and 200 µg/mL at 28 °C) were based on a preliminary study with limited test doses and fish samples which resulted in the selection of different test doses between the two temperatures. Unfortunately, the difference in the selected doses prevents us from making a direct comparison of the drug action between the two temperatures at the same dose level. Despite this imperfection, it was clear that the optimal doses of MS-222 in Asian seabass at 22 and 28 °C were surprisingly similar (140 and 150 µg/mL, respectively) given that there was a big difference in the rearing water temperature. Nonetheless, it should be mentioned that the determined optimal doses of MS-222 in Asian seabass in the current study were higher than those reported formerly, which was 70–90 µg/mL at 25 °C [16]. One reason for this inconsistency might be due to the size difference in the fish. In our previous study with marketable-size Nile tilapia (400–600 g), the determined optimal doses of MS-222 (300 µg/mL) and eugenol (90 µg/mL) [22] were higher than the doses for tilapia fries (3 g), which were 105 and 20 µg/mL, respectively [35]. Likewise, the effective doses of MS-222 for anesthetizing large-size (200–1300 g) and small-size lumpfish (10–20 g) were 200 and 100 µg/mL, respectively [21], while the appropriate dose for lumpfish fries (1 g) was only 60 µg/mL [29]. Nevertheless, it is worth mentioning that the relationship between a fish’s body weight and anesthetic action is not always straightforward [8,36].

For reaching stage III anesthesia, Asian seabass immersed with a higher MS-222 dose had a shorter induction time, which agreed with several studies [8,16,21,22,35], but the effect on recovery time was often more variable. Regarding the MEC, at a given temperature level, similar MECs were found within three different doses as reported in our previous paper with Nile tilapia [22]. The comparable MEC across different doses was most likely responsible for the observed faster induction time when the higher MS-222 dose was used. Comparing the MECs of MS-222 among different fish species, the drug’s MEC in Asian seabass had a similar level to rainbow trout, northern pike, silver seabream, and Nile tilapia [11,20,22] but fivefold higher than Atlantic salmon [24]. These results emphasize the importance of testing the anesthetic doses on each species for managing the fish in a proper way.

Inducing Asian seabass to stage III anesthesia with MS-222 did change some physiological parameters, as revealed by the blood biochemical study. When a fish is anesthetized, physiological disturbances like hypoventilation are generally followed, which leads to temporary hypoxia [37]. The hypoxic condition following MS-222 application in this study was indicated by the increased blood pCO_2_ as also seen in yellow perch (*Perca flavescens*), walleye pike (*Sander vitreus*), and rainbow trout [38,39]. The blood pH is decreased at the same time, because the accumulation of carbon dioxide increases the acidity of the blood [40]. Under hypoxic condition, the production of lactate is increased as an energy substrate for anaerobic respiration [41,42], which was the case in this study and short-horned sculpin (*Myoxocephalus scorpius*) [43]. It has been previously discovered that the increase in lactate level goes back to normal at 4 h post-anesthetic exposure in bonefish (*Albula vulpes*) and Atlantic salmon [41,44]. Many researchers have reported that exposure to high-concentration anesthetics elevated the blood glucose level, indicating the occurrence of stress [38,41,45,46]. After anesthetic exposure, the hypoxia state in fish may trigger the releasing of catecholamines then stimulate glucose production subsequently [47,48]. On juveniles of matrinxã (*Brycon cephalus*), dose-dependent changes in glucose occur with MS-222 exposure [49], which again highlights the necessity of using optimal dose while conducting fish anesthesia to ensure safety. In addition, the level of magnesium in blood was raised after induction with MS-222, which also happens in brook trout (*Salvelinus fontinalis*) [50]. Among other electrolyte parameters, the calcium, sodium, and chloride levels did not change significantly by anesthesia, which agrees with the findings of Parker-Graham et al., who studied koi anesthesia [51]. The increased potassium levels observed in this study may be related to the inhibition of action potential under anesthetic condition and subsequent muscle relaxation. [12,13].

Due to the difference in the determined optimal doses between 22 and 28 °C, the effect of temperature could not be unequivocally assessed for the dose-dependent PK parameters including AUC. On the contrary, for the dose-independent PK parameters such as t_1/2λ_ and MRT, the influence of water temperature was evident. The fact that the t_1/2λ_ and MRT of MS-222 were shortened at 28 °C compared to 22 °C reflects the faster drug elimination at the warmer temperature, which is comprehensible since the metabolic rate is generally increased at higher temperatures [30,52]. Although the enhanced drug elimination at higher temperatures has been widely reported in several antimicrobial drugs in the literature [32,33,34,53], similar information on MS-222 is rare. Nevertheless, it is clear that drug elimination usually shows a positive correlation with water temperature regardless of drug class.

Following MS-222 immersion, the drug was easily absorbed via the gill into the bloodstream due to its high lipid solubility and then crossed the blood–brain barrier to reach the central nervous system [3,12,14], so higher concentrations of MS-222 were found in the brain as early as 5 min after reaching stage III anesthesia at both temperatures. In addition to the brain, high concentrations of MS-222 were also detected in the liver, gill, and kidney when compared with the muscle. The result was consistent with another similar study that reported a higher MS-222 concentration in the liver of Asian seabass than in the muscle tissue [16]. Given that the brain, liver, gill, and kidney are highly vascularized, whereas the muscle contains fewer blood vessels, it is understandable that the drug would be well distributed via the systemic circulation into the former tissues rather than the latter. Note that the tissue distribution pattern was in agreement with the serum concentration–time profile of MS-222 (Figure 1), which exhibited a characteristic biphasic depletion (bi-exponential decay) on a semi-logarithmic scale, suggesting that MS-222 may be characterized by a two-compartmental model [54,55]. To confirm this speculation, more blood sampling time points are needed, especially at the early phase (i.e., distribution phase) of drug depletion. It is also interesting to note that with the tissue distribution study, a clearer picture regarding the overall drug movement in the fish can be derived. For instance, the brain and gill showed peak concentration at the first sampling time point, while the liver and kidney peaked at later times (second and third time points). The muscle exhibited a similar pattern but was followed by a much slower elimination phase at significantly lower drug concentrations. It is generally recognized that fish predominantly discharge anesthetic substances via the gill [24,56]. The fact that relatively constant MS-222 concentrations (3–4 µg/g) were found in the gill after the Asian seabass had been transferred into the recovery tanks for 10–90 min also supports the assumption that the gill was the main route for MS-222 excretion. In addition to the gill excretion, the elimination of MS-222 also relies on hepatic biotransformation and renal excretion but possibly to a lesser extent [57,58]. These observations, when combined together, give rise to a sensible route of drug movement in and out of the respective organs for drug action, distribution, and elimination in Asian seabass. Nevertheless, even though MS-222 rapidly depleted in most tissues of Asian seabass within 90 min, the safe withdrawal time for human consumption remains to be investigated in a future study using the standard linear regression analysis.

## 5. Conclusions

Water temperature has minimal effects on the optimal doses and MECs of MS-222 in Asian seabass. Irrespective of rearing temperature, fish exposed to MS-222 resulted in significant changes in blood lactate, glucose, pH, and ions including calcium, magnesium, and sodium. Potassium and pCO_2_ were significantly changed only at 22 °C, whereas chloride and anion gap only at 28 °C. After termination of MS-222 immersion at the optimal dose, the drug concentrations in the gill were maintained throughout the experimental period, while most other tissues declined progressively with time. The dose-independent PK parameters such as t_1/2λ_ and MRT were shortened at 28 °C, indicating faster drug elimination at the warmer temperature. The serum half-lives were reduced by half (37.01 h vs. 18.43 h) at 28 °C. The study provides ample physiological and pharmacokinetic evidence, contributing to a better understanding of the actions of MS-222 in Asian seabass at different temperatures.

## Figures and Tables

**Table 1:** The determinant criteria and minimum effective concentration (MEC) of tricaine methanesulfonate (MS-222) in Asian seabass at 22 and 28 °C following single immersion administrations (*n* = 7).

Temperature	Dose	Induction Time	Induction Time < 5 min (Fish Number)	Recovery Time	Recovery Time < 5 min (Fish Number)	Minimum Effective Concentration (MEC)	Grand Mean of MEC
(°C)	(µg/mL)	(min)		(min)		(µg/mL)	(µg/mL)
22	100	16.41 ± 1.80 ^a^	0/7	2.68 ± 1.30 ^a^	7/7	58.20 ± 12.22 ^a^	
	140	4.17 ± 0.45 ^b^	7/7	2.95 ± 1.04 ^a^	7/7	74.44 ± 24.61 ^a^	70.48 ± 21.56
	175	2.64 ± 0.60 ^c^	7/7	5.02 ± 1.02 ^b^	4/7	78.80 ± 22.80 ^a^	
28	100	29.37 ± 3.81 ^a^	0/7	2.77 ± 1.43 ^a^	7/7	76.84 ± 8.57 ^a^	
	150	3.25 ± 0.83 ^b^	7/7	2.80 ± 0.30 ^a^	7/7	76.03 ± 15.33 ^a^	78.27 ± 16.22
	200	1.32 ± 0.35 ^c^	7/7	3.92 ± 0.98 ^a^	6/7	81.96 ± 23.34 ^a^	

Note: Data presented are mean ± SD. For each temperature level, means with different superscripts in each column are significantly different from each other (*p* < 0.05).

**Table 2:** The blood parameters of Asian seabass via blood chemistry analysis and blood gas analysis before and after tricaine methanesulfonate (MS-222) immersion at the optimal doses at 22 °C (140 µg/mL) and 28 °C (150 µg/mL) (*n* = 7).

Parameters	Unit	Temperature			
		22 °C		28 °C	
		Before	After *	Before	After *
Lactate	mmol/L	0.50 ± 0.00 ^a^	1.36 ± 0.61 ^b^	0.52 ± 0.06 ^a^	2.29 ± 0.91 ^b^
Glucose	mg/dL	30.71 ± 3.25 ^a^	104.43 ± 35.43 ^b^	29.14 ± 1.86 ^a^	106.57 ± 55.78 ^b^
BUN	mg/dL	2.43 ± 1.13 ^a^	2.43 ± 1.13 ^a^	1.86 ± 0.90 ^a^	2.14 ± 1.07 ^a^
Calcium	mg/dL	9.64 ± 0.21 ^a^	10.24 ± 0.52 ^b^	9.77 ± 0.08 ^a^	10.64 ± 0.40 ^b^
Magnesium	mg/dL	2.76 ± 0.15 ^a^	3.16 ± 0.32 ^b^	2.63 ± 0.16 ^a^	3.10 ± 0.16 ^b^
Sodium	mEq/L	166.14 ± 2.61 ^a^	170.14 ± 3.18 ^b^	165.43 ± 3.26 ^a^	171.86 ± 3.44 ^b^
Potassium	mEq/L	3.57 ± 0.43 ^a^	2.93 ± 0.39 ^b^	4.31 ± 0.83 ^a^	4.06 ± 1.33 ^a^
Chloride	mEq/L	145.71 ± 1.80 ^a^	147.29 ± 2.50 ^a^	146.57 ± 1.72 ^a^	149.14 ± 2.48 ^b^
pH		7.69 ± 0.02 ^a^	7.62 ± 0.05 ^b^	7.57 ± 0.04 ^a^	7.48 ± 0.10 ^b^
HCO_3_^−^	mmol/L	9.16 ± 0.79 ^a^	9.86 ± 0.85 ^a^	8.89 ± 1.04 ^a^	8.84 ± 1.90 ^a^
pCO_2_	mmHg	7.14 ± 0.69 ^a^	9.14 ± 0.69 ^b^	9.57 ± 1.40 ^a^	11.29 ± 1.80 ^a^
tCO_2_	mmol/L	9.60 ± 0.82 ^a^	10.40 ± 0.85 ^a^	9.30 ± 1.12 ^a^	9.37 ± 1.92 ^a^
Anion gap	mEq/L	22.95 ± 1.33 ^a^	23.38 ± 1.19 ^a^	22.86 ± 1.41 ^a^	24.91 ± 1.55 ^b^

Note: * The blood samples were collected soon after the fish reached anesthesia (stage III). The data are presented as mean ± SD. BUN, blood urea nitrogen; HCO_3_^−^, bicarbonate ion; pCO_2_, partial pressure of carbon dioxide; tCO_2_, total carbon dioxide. Data presented are mean ± SD. For each temperature level, means with different superscripts in each row are significantly different from each other (*p* < 0.05).

**Table 3:** Pharmacokinetic parameters of tricaine methanesulfonate (MS-222) in Asian seabass after 3 min immersion at the optimal doses at 22 °C (140 µg/mL for 3 min) and 28 °C (150 µg/mL for 3 min) (*n* = 8).

Parameters	Unit	Temperature	
		22 °C	28 °C
λ	1/min	0.019	0.038
t_1/2 λ_	min	37.01	18.43
AUC	min·µg/mL	304.48	306.68
MRT	min	28.43	14.27

Note: The pharmacokinetic parameters of serum MS-222 were determined by a non-compartmental model. λ, terminal rate constant; t_1/2λ_, terminal half-life; AUC, area under the serum concentration–time curve; MRT, mean residence time.

## Data Availability

Data sharing not applicable.
